# Secondhand smoke exposure at home among middle and high school students in the United States – does the type of tobacco product matter?

**DOI:** 10.1186/s12889-017-4019-z

**Published:** 2017-01-19

**Authors:** Florian Fischer, Alexander Kraemer

**Affiliations:** Department of Public Health Medicine, School of Public Health, Bielefeld University, P.O. Box 100 131, 33501 Bielefeld, Germany

**Keywords:** Secondhand smoke, National Youth Tobacco Survey, United States, USA, Students

## Abstract

**Background:**

A decline in the prevalence of secondhand smoke (SHS) exposure has been observed in the United States of America (USA) during the past few decades. Nevertheless, nearly half of non-smoking students are still exposed to SHS. This paper aims to describe the factors associated with SHS exposure stratified by type of exposure (overall, cigarettes and electronic cigarettes).

**Methods:**

The analysis is based on secondary data taken from the National Youth Tobacco Survey 2014. Overall, 22,007 middle and high school students from the USA are included in the sample. Descriptive and bivariate statistics as well as binary logistic regression models were performed.

**Results:**

Overall, 44.5% (*n=*9,798) of the study participants declared themselves to be exposed to SHS, 29.1% (*n=*6,394) declared to be exposed to SHS caused by cigarette smoke and 9.4% (*n=*2,067) claimed that a person who lives with them uses electronic cigarettes. There is a considerable overlap between the two types of SHS exposure, because 74.9% (*n=*1,548) of students declaring that a person within their household uses electronic cigarettes also declare a person in the household smoking cigarettes. The strengths of association between independent variables and SHS exposure differs by type of exposure and also by smoking status of respondents.

**Conclusions:**

Although only small differences are obvious in the factors associated with SHS exposure stratified by the type of tobacco product, there are still some variations which should be considered in policy making to allow for a targeted approach in prevention campaigns or legislation.

## Background

Tobacco use causes an estimated 480,000 deaths per year in the United States of America (USA); almost 10% of these deaths are attributable to secondhand smoke (SHS) exposure among non-smokers [1]. SHS exposure is associated with serious health problems, especially in children [2]. Declines in the prevalence of self-reported SHS exposure during the past decades have been observed in the US in children, adolescents and adults [3, 4]. Nevertheless, data from students (grades 6 to 12) in the USA have shown that nearly half of non-smoking students were exposed to SHS in at least one location in 2013 [5].

In addition to the still high level of SHS exposure, more attention has to be paid to the growing popularity of electronic cigarettes (e-cigarettes). Electronic cigarettes are battery-powered devices capable of delivering nicotine and other additives (e.g., flavorings) to the user in an aerosol form [6, 7]. Recent evidence suggests that electronic cigarettes may be overtaking conventional cigarettes in popularity [8, 9]. In 2014, electronic cigarettes became the most commonly used tobacco product among middle and high school students in the USA [8, 10]. Although the popularity and use of electronic cigarettes is continuing to increase, there is a lack of data on the exposure and potentially adverse health effects attributable to both their use and the SHS exposure caused by electronic cigarettes [6, 11–14]. The vapor from electronic cigarettes, which is a type of SHS exposure for people standing nearby smokers, also exposes non-smokers to contaminants, including nicotine, particulates and hydrocarbons. However, the health risks appear to be lower than from SHS exposure caused by other tobacco products [15, 16].

Although the main concerns with electronic cigarettes are related to their effects on smokers, the effects on non-smokers inhaling the vapor also have to be considered. Furthermore, electronic cigarettes may have the potential to become a gateway to tobacco use, because they may lead to a renormalization and social acceptance of smoking [17–23]. Discussions about the role of electronic cigarettes in tobacco initiation among teens have recently begun to develop [22, 24]. One current study has already indicated that the association between electronic cigarettes and smoking initiation may be stronger among younger than older children [25].

The high levels of SHS exposure, despite an overall decreasing trend, as well as the increasing use of electronic cigarettes, pose several challenges to public health and policy makers. For that reason, this paper aims to describe the factors associated with SHS exposure at home among middle and high school students in the USA. Students are an important subgroup for public health activities, because many health and risk behaviors are developed in young ages. Bad habits which may affect the whole lifespan are frequently coined in this phase. Therefore, the knowledge of factors associated with SHS exposure are necessary if we are to develop and implement adequate and target group specific strategies to protect non-smokers from SHS exposure. The aims of this analysis are 1) to estimate the SHS exposure prevalence (overall, cigarettes and electronic cigarettes), 2) to investigate the factors associated with SHS exposure, and 3) to evaluate whether the association is the same depending on the type of SHS exposure.

## Methods

### Data source

The analysis is based on secondary data taken from the National Youth Tobacco Survey (NYTS) 2014. This survey aims to provide the necessary data to support the design, implementation and evaluation of smoking prevention and control programs in the USA. The NYTS provides a nationally representative sample of middle and high school students in the USA. A stratified, three-stage cluster sample design was applied to select schools for participation. Overall, 207 schools located in 36 states of the USA participated in the survey (out of 258 selected during the probabilistic sampling procedure). The exclusion of incomplete questionnaires leads to a sample of 20,007 students. Participation in the survey was voluntary. The student participation rate among participating schools was 91.4%. The overall participation rate, defined as the product of school-level and student-level participation rates, was 73.3%. Data was collected by trained data collectors during February to June 2014. Students completed a self-administered questionnaire containing 81 questions using paper and pencil [26].

### Variables selected for analysis

Three dependent variables were chosen for the analysis: 1) Current overall SHS exposure at home: This variable was based on the question, if anyone who lives with the study participant uses any form of tobacco at the time of the interview. The question was: “Does anyone who lives with you now…?”. It contains several sources of SHS (cigarettes; cigars, cigarillos, little cigars; chewing tobacco, snuff, dip; electronic cigarettes; hookah, waterpipe; pipes filled with tobacco; snus; dissolvable tobacco products; bidis) and the respondent was able to provide multiple answers. To allow for comparisons between cigarettes, a highly prevalent source of SHS exposure, and electronic cigarettes, a newly emerging source of SHS exposure, those two sources were considered in particular: 2) Current exposure to cigarette smoke at home: This variable was based on the same question, except that the exposure was restricted to cigarettes only. 3) Current exposure to electronic cigarettes by a person who lives with the respondent. The outcomes were binary (“yes” vs. “no”) for the three outcomes [26].

The selection of independent variables associated with SHS exposure was literature-based. Since the analysis is based on secondary date, the inclusion of variables was dependent on each variable’s availability in the data set. Age was categorized into four groups (“9–12 years”, “13–15 years”, “16–17 years” and “18 years and above”). Sex was included as another demographic variable. Education (in terms of grade) was not included, because this is highly correlated with the age of students.

Several variables were selected which may be associated with SHS exposure. Among them, own smoking behavior was assessed by two approaches: 1) The question which tobacco product the students tried first was used to provide information on whether the students “tried smoking” (which was coded if one tobacco product was mentioned by the student) or “never tried smoking”. 2) In addition, current smoking behavior in the past 30 days was assessed (“smoking” vs. “not smoking”).

Reactions towards a friend offering a cigarette (“starting to smoke” vs. “not starting to smoke”) were used as a proxy for the influence of the social environment on how smoking was judged by respondents. Students were asked how they judge the harms of smoking a cigarette. We used a binary outcome for the interpretation of the harms (“no or little harm” vs. “a lot of harm”). Furthermore, a critical and proactive consideration of the harmful effects was assessed by posing the question if the respondent has thought about the harmful chemicals in tobacco products; the answers were classified into three categories (“rarely or never”, “sometimes” and “often or very often”). In addition, respondents were asked how often they see advertisements for cigarettes and other tobacco products on the internet (“rarely or never”, “sometimes” and “most of the time”).

### Statistical analyses

All statistical analyses were performed using the statistical software package IBM SPSS Statistics 23. The complex survey analysis routine, as described in the NYTS methodology report [26], was used for data analysis, by estimating variances using the method of linearized estimators. Firstly, frequency runs were explored to present descriptive information about the sample (including percentages and means). These sample size characteristics as well as bivariate analyses in terms of cross tables were presented without using a weighting factor. For the logistic regression models a weighting factor was used to account for non-response and for varying probabilities of selection. The weights were adjusted to ensure that the weighted proportions of students in each grade matched national population proportions. This weighting factor was provided with the data set.

Cross tables between the three dependent variables and all independent variables were performed to explore the associations between SHS exposure (overall and specific to cigarettes or electronic cigarettes) and nominal or ordinal scaled independent variables. We used the Chi-square test (χ^2^) of independence to analyze the associations of two variables with multiple categories. All tests were two-sided and statistical significance was based on an alpha-level of 0.05. Comparatively small intercorrelations between independent variables and low Variance Inflation Factors (VIF) – ranging from 1.011 to 1.328 for the variables selected for the binary logistic regression models – indicated no multicollinearity.

Finally, six binary logistic regression models were calculated to highlight the associations between SHS exposure (SHS exposure overall, SHS exposure due to cigarettes and SHS exposure due to electronic cigarettes), stratified by smoking status (ever vs. never), and several independent variables. We calculated odds ratios (OR) and 95% confidence intervals (CI) for SHS exposure compared to no exposure. Nagelkerke’s R^2^ was calculated to provide an overview of the variance explained by the variables used in the regression models.

## Results

### Descriptive and bivariable analyses

The characteristics of the sample are described in Table 1, along with the level of exposure to SHS at home. Overall, 44.5% (*n=*9,798) of the study participants declared themselves to be exposed to SHS, 29.1% (*n=*6,394) declared to be exposed to SHS caused by cigarette smoke and 9.4% (*n=*2,067) claimed that a person who lives with them uses electronic cigarettes. There is a considerable overlap between the two types of SHS exposure, because 74.9% (*n=*1,548) of students declaring that a person within their household uses electronic cigarettes also declare a person in the household smoking cigarettes. This analysis indicates that exposure to cigarettes seems to be the main factor in SHS exposure. These two outcome variables are highly correlated (*r=*0.714; *p*<0.001), which is obviously due to the fact that all students who declare being exposed to cigarette smoke by a person who lives with them also have to declare SHS exposure in general. Only a moderate correlation was observed between SHS exposure in general and exposure to SHS due to electronic cigarettes (*r=*0.359; *p<*0.001).

**Table 1:** Sample characteristics and SHS exposure at home, USA 2014^a^

	*n* (%)	SHS exposure overall *n* (%)	SHS exposure due to cigarettes *n* (%)	SHS exposure due to electronic cigarettes *n* (%)
Age		*p =* 0.350	*p =* 0.548	*p =* 0.221
9–12 years	4,461 (20.4)	1,936 (43.4)	997 (24.4)	113 (3.5)
13–15 years	9,774 (44.7)	4,338 (44.4)	2,125 (24.2)	245 (3.5)
16–17 years	5,753 (26.3)	2,571 (44.7)	1,259 (24.2)	127 (3.1)
18+ years	1,862 (8.5)	851 (45.7)	440 (25.8)	33 (2.5)
Sex		*p =* 0.462	*p =* 0.017	*p =* 0.226
Male	11,150 (51.2)	4,921 (44.1)	2,402 (23.6)	282 (3.5)
Female	10,645 (48.8)	4,751 (44.6)	2,401 (25.1)	233 (3.1)
Usage of tobacco products		*p <* 0.001	p < 0.001	p < 0.001
Tried smoking	6,591 (30.6)	4,029 (61.1)	1,844 (33.9)	262 (6.8)
Never tried smoking	14,932 (69.4)	5,396 (36.1)	2,950 (21.0)	251 (2.2)
Cigarette smoking status (past 30 days)		p < 0.001	p < 0.001	p < 0.001
Smoking	1,392 (6.5)	999 (71.8)	463 (42.8)	44 (6.6)
Not smoking	20,108 (93.5)	8,447 (42.0)	4,285 (23.3)	461 (3.2)
Reaction to best friend offering a cigarette		*p <* 0.001	*p <* 0.001	*p <* 0.001
Starting to smoke	2,492 (11.4)	1,633 (65.5)	717 (35.7)	86 (6.2)
Not starting to smoke	19,336 (88.6)	8,034 (41.5)	4,095 (23.0)	426 (3.0)
Judgement of harm caused by smoking		*p <* 0.001	*p <* 0.001	*p <* 0.001
No or little harm	10,241 (47.9)	4,887 (47.7)	2,470 (27.1)	276 (4.0)
A lot of harm	11,140 (52.1)	4,449 (39.9)	2,325 (22.7)	238 (2.9)
Thought about harmful chemicals in tobacco products		*p <* 0.001	*p <* 0.001	*p <* 0.001
Rarely or never	14,088 (66.7)	5,639 (40.0)	2,955 (22.9)	322 (3.1)
Sometimes	4,040 (19.1)	1,838 (45.5)	988 (27.5)	102 (3.8)
Often or very often	2,990 (14.2)	1,590 (53.2)	848 (32.9)	91 (5.0)
Receiving advertisements for tobacco products on the internet		p < 0.001	*p <* 0.001	*p <* 0.001
Rarely or never	14,088 (51.6)	4.251 (40.0)	2,244 (23.1)	239 (3.1)
Sometimes	4,040 (33.4)	2.978 (43.4)	1.583 (25.6)	163 (3.4)
Most of the time	3,085 (15.0)	1.672 (54.2)	835 (31.5)	97 (5.1)

^a^ *p*-values based on chi-square test or Fisher’s exact test

Almost no differences in exposure are observed in the four age groups, although SHS exposure due to electronic cigarettes is slightly higher in younger age groups. Females are slightly more frequently exposed than males, except for electronic cigarettes. Students who have already tried to smoke show a higher prevalence of SHS exposure. In addition, students who claim they started to smoke after one of their best friends offered them a cigarette are much more likely to be exposed to SHS. Students judging the harms of cigarette smoke to be more severe show lower levels of SHS exposure. In contrast, students who think about the harmful chemicals in tobacco products more frequently indicate a higher level of SHS exposure. Also, a higher perception of advertisements for tobacco products on the internet is associated with higher exposure to SHS.

### Binary logistic regression models

The results of binary logistic regression models are shown in Table 2. The results are stratified by smoking behavior as well as type of SHS exposure (overall, cigarettes or electronic cigarettes). The most significant associations were found for the relationship between the independent variables and SHS exposure overall. The risk of SHS exposure is lowest in the group of students aged 18 years and above. The lower the age, the higher the risk of SHS exposure in all types of exposure – except for students aged 16–17 years exposed to SHS caused by cigarettes. SHS exposure due to electronic cigarettes in the group of students who never tried any kind of tobacco product was not significantly associated with age. In contrast, the ORs for the younger age categories were quite high in students who tried a tobacco product before.

**Table 2:** Binary logistic regression models for factors associated with SHS exposure at home, USA 2014^a^

	Never tried any kind of tobacco product			Tried any kind of tobacco product		
	SHS exposure overall OR (95% CI) *n=*14,932	SHS exposure due to cigarettes OR (95% CI) *N=*14,056	SHS exposure due to electronic cigarettes OR (95% CI) *N=*11,357	SHS exposure overall OR (95% CI) *N=*6,591	SHS exposure due to cigarettes OR (95% CI) *N=*5,447	SHS exposure due to electronic cigarettes OR (95% CI) *N*=3,865
Age (ref.: 18+ years)						
9–12 years	1.26 (1.06–1.48)	1.15 (0.94–1.40)	1.69 (0.84–3.42)	2.08 (1.62–2.68)	1.49 (1.14–1.96)	4.37 (2.48–7.69)
13–15 years	1.15 (0.98–1.35)	1.02 (0.85–1.23)	1.68 (0.85–3.32)	1.60 (1.36–1.88)	1.17 (0.97–1.40)	2.07 (1.29–3.32)
16–17 years	1.05 (0.89–1.25)	0.91 (0.74–1.11)	1.12 (0.54–2.33)	1.08 (0.92–1.26)	0.91 (0.76–1.08)	1.36 (0.84–2.20)
Sex (ref.: female)						
Male	0.91 (0.84–0.97)	0.96 (0.88–1.05)	1.22 (0.94–1.58)	0.87 (0.78–0.97)	0.82 (0.73–0.92)	0.86 (0.66–1.12)
Reaction to best friend offering a cigarette (ref.: not starting to smoke)						
Starting to smoke	1.40 (1.11–1.77)	1.10 (0.83–1.46)	1.13 (0.49–2.58)	1.46 (1.30–1.65)	1.31 (1.15–1.50)	1.03 (0.76–1.39)
Judgement of harm caused by smoking (ref.: a lot of harm)						
No or little harm	1.30 (1.21–1.40)	1.28 (1.17–1.39)	1.47 (1.13–1.91)	1.24 (1.11–1.38)	1.15 (1.02–1.31)	1.15 (0.87–1.52)
Thought about harmful chemicals in tobacco products (ref.: often or very often)						
Rarely or never	0.63 (0.57–0.70)	0.62 (0.55–0.70)	0.66 (0.45–0.95)	0.64 (0.55–0.74)	0.68 (0.58–0.80)	0.76 (0.53–1.10)
Sometimes	0.75 (0.66–0.85)	0.77 (0.67–0.89)	0.71 (0.45–1.11)	0.76 (0.64–0.91)	0.80 (0.66–0.97)	1.02 (0.68–1.55)
Receiving advertisements for tobacco products on the internet (ref.: most of the time)						
Rarely or never	0.61 (0.55–0.68)	0.67 (0.59–0.76)	0.59 (0.41–0.85)	0.73 (0.62–0.84)	0.79 (0.67–0.93)	0.78 (0.54–1.11)
Sometimes	0.69 (0.62–0.78)	0.74 (0.65–0.84)	0.63 (0.43–0.93)	0.77 (0.66–0.90)	0.88 (0.74–1.05)	0.83 (0.57–1.21)
R^2^	2.5%	1.8%	1.5%	4.7%	2.6%	3.2%

^a^weighted results

Females showed a higher chance of being exposed to SHS than males, but this was also significant only for SHS exposure overall and SHS exposure due to cigarettes in students who tried any kind of tobacco product. Attributing only a little or even no harm to smoking is associated with a 15 to 47% increase in the likelihood of being exposed to SHS. But this association was not significant for SHS exposure due to electronic cigarettes in students having tried to smoke themselves. Furthermore, the results indicate that perceiving advertisements for tobacco products is associated with a higher chance of being exposed to SHS. This association is not that for SHS exposure due to electronic cigarettes.

The results are comparable overall for the three outcomes, but a higher variance, based on the results of Nagelkerke’s R^2^, can be explained for SHS exposure overall than for SHS exposure caused by electronic cigarettes or cigarettes in particular.

## Discussion

The study highlights the relevance of SHS exposure at home, because nearly half of students still declare that at least one person in the household uses tobacco products, which may be seen as a proxy for SHS exposure. There are different types of SHS exposure, depending upon the tobacco product used. In recent times, electronic cigarettes have joined the field as a source of SHS exposure. Despite the increasing use of electronic cigarettes, which were supposed to overtake conventional cigarettes in popularity [8, 9], only 9.4% of study participants declared themselves to be exposed to electronic cigarettes, compared to 29.1% being exposed to SHS caused by cigarette smoke. The overall SHS exposure (44.5%) is comparable to results presented from NYTS in 2009 (40.5%) [3], but the former declining trend was not continued.

Current research indicates that there is no or only low harm caused by electronic cigarettes to persons standing nearby. A variety of organic chemicals were detected, but electronic cigarette use does not measurably increase the quantities of these chemical substances above background levels, whereas SHS exposure caused by cigarettes, for example, increases these levels considerably [15, 16]. Nevertheless, the long-term effects of electronic cigarettes are not yet understood. Furthermore, perceiving electronic cigarettes as less harmful may motivate non-smokers to use them. Therefore, much more research focusing on the perception of risk of electronic cigarettes on health and risk behaviors is needed [27].

Hammal and Finegan [28] indicated that young people and adolescents were more willing to use electronic cigarettes under peer influence than conventional cigarettes, because electronic cigarettes were perceived as less harmful. The results of our study highlight that thinking less about the harmful chemicals in tobacco products is associated with a lower OR for SHS exposure. This might be explained by the fact that it is particularly smokers or people being exposed to SHS who think about these harmful effects, whereas they are irrelevant to non-smokers and people who are not exposed to SHS.

Although only small differences are observed in the factors associated with SHS exposure stratified by the type of tobacco product, there are still some variations which should be considered in policy making to allow for a targeted approach in prevention campaigns or legislation. Gilreath et al. [29] have already mentioned the need for tobacco control interventions which address specific tobacco products.

Legislation for the protection of non-smokers may be one useful approach to tobacco control. In recognition of the adverse health effects caused by SHS exposure, the US Department of Housing and Urban Development (HUD) issued a proposed rule in November 2015 that will prohibit indoor smoking in HUD-supported public housing properties [30]. This action applies to all types of SHS exposure. With respect to electronic cigarettes, several pieces of legislation have already been passed in the USA [31, 32]. For example, one-fifth of US states permitted the sale of electronic cigarettes to children in early 2015 [33].

Nevertheless, more efforts are still needed to protect particularly vulnerable populations, such as the students who are the focus of this study. Further approaches, like raising awareness [34], for example through education and media campaigns, or raising taxes, are necessary to protect non-smokers at home as well as in other settings. In this context, Leone et al. [35] referred to an advertisement against smoking: “There’s a warning label on cigarette packs for people who smoke. Where should the warning go for people who breathe?” This emphasizes the main advantage of campaigns aiming to protect non-smokers’ health. Therefore, prohibition or reduction of advertisements for tobacco products is needed.

Our study results indicate that, for all types of SHS exposure, seeing no or few advertisements for tobacco products on the internet is associated with a lower chance of being exposed to SHS. In this context, it is not only advertisements for cigarettes that have to be banned, but also advertisements for electronic cigarettes. An analysis by Singh et al. [7] showed that nearly 70% of middle and high school students in the USA were exposed to electronic cigarette advertisements from at least one source. This high prevalence confirms the relevance of reducing these kinds of advertisements. Furthermore, clear and unambiguous information about the risks of being exposed to SHS or the vapor from electronic cigarettes is required [28].

### Limitations

The analyses in this study are based on a national and representative sample of adolescents in the USA. The data collected in the NYTS is based on a standardized questionnaire and sampling frame. The CIs are very small for all variables, which indicates precision in the results. But the large sample size, which is reinforced by the weighting factor, also has to be considered in this regard.

The interpretation of results faces several limitations. Firstly, it is a self-administered questionnaire. Therefore, possible recall bias may result in an under-reporting of indoor SHS exposure. Although the response rate is high, there might be some bias due to the fact that the data applies only to participating schools and youths. Students who refused to participate or did not attend school were not included in the analysis. Secondly, only SHS exposure at home was considered. For this reason, various further locations where students might be exposed to SHS could not be assessed. In addition, SHS exposure was only assessed by the question whether someone in the household uses various types of tobacco products (such as cigarettes and electronic cigarettes). This does not necessarily mean that SHS reaches the respondent. For that reason, this question can only be used as a proxy for SHS exposure, because no validation of self-reports was conducted [36]. Thirdly, the data is cross-sectional. Therefore, only associations and not causalities can be displayed. Fourthly, the overall explained variance is quite low. Due to data limitations, we are unable to include more variables. In particular the limited range of sociodemographic variables and missing information on socioeconomic or family background leads to major limitations in the modeling approach. For example, it is well described that SHS exposure occurs more frequently in households with lower socioeconomic levels, which is, therefore, a highly important determinant that could not be included in the analysis. Fifthly, the analysis is only stratified by overall SHS exposure and SHS exposure caused by cigarettes or electronic cigarettes. Further tobacco products were not included due to the low prevalence of SHS exposure due to such products. Sixthly, exposure to SHS may also be strongly associated with the characteristics of people who smoke at home (e.g. the sex, age and education of parents or people living with the student). This information was not provided in the data set and, therefore, could not be included in the analysis. In addition, no information on geographical location was available. Therefore, the effects of smoke-free policies or other tobacco-control policies, which vary between states, could not be taken into account.

## Conclusions

Despite a steady decline in SHS exposure, it is still a relevant risk factor due to the high prevalence of exposure, the great vulnerability of children, and the increasing popularity and use of electronic cigarettes. The study results highlight that nearly half of middle and high school students are exposed to SHS at home. SHS may not only impact upon health due to direct effects caused by contaminants, but also due to indirect factors such as the renormalization of smoking within a population. Therefore, the growing importance of electronic cigarettes needs to be considered in future measures designed for the protection of non-smokers. Electronic cigarettes and SHS exposure may be a mediator between parental smoking and smoking initiation among children [37, 38]. The study indicates that the factors associated with SHS exposure differ only slightly after they are stratified in terms of the type of tobacco product leading to SHS exposure. Nevertheless, these variations have to be considered when developing and implementing tobacco control strategies to protect non-smokers.

## Abbreviations

CI: Confidence interval
HUD: US Department of Housing and Urban Development
NYTS: National Youth Tobacco Survey
OR: Odds ratio
SHS: Secondhand smoke
USA: United States of America
